# Enhancement of Stability Towards Aging and Soil Degradation Rate of Plasticized Poly(lactic Acid) Composites Containing Ball-Milled Cellulose

**DOI:** 10.3390/polym17152127

**Published:** 2025-08-01

**Authors:** Roberta Capuano, Roberto Avolio, Rachele Castaldo, Mariacristina Cocca, Federico Olivieri, Gennaro Gentile, Maria Emanuela Errico

**Affiliations:** 1 Institute of Polymers, Composites and Biomaterials, National Research Council of Italy (IPCB-CNR), Via Campi Flegrei 34, 80078 Pozzuoli, Italy; roberta.cap2012@gmail.com (R.C.); rachele.castaldo@cnr.it (R.C.); mariacristina.cocca@cnr.it (M.C.); federico.olivieri@cnr.it (F.O.); gennaro.gentile@cnr.it (G.G.); 2 Department of Mechanical and Industrial Engineering—DIMI, University of Brescia, Via Branze 38, 25121 Brescia, Italy

**Keywords:** mechanochemistry, plasticization, PLA, biodegradation, biocomposites

## Abstract

In this study, multicomponent PLA-based biocomposites were developed. In particular, both native fibrous cellulose and cellulose with modified morphology obtained through ball milling treatments were incorporated into the polyester matrix in combination with an oligomeric plasticizer, specifically a lactic acid oligomer (OLA). The resulting materials were analyzed in terms of their morphology, thermal and mechanical properties over time, water vapor permeability, and degradation under soil burial conditions in comparison to neat PLA and unplasticized PLA/cellulose composites. The cellulose phase significantly affected the mechanical properties and enhanced their long-term stability, addressing a common limitation of PLA/plasticizer blends. Additionally, water vapor permeability increased in all composites. Finally, the ternary systems exhibited a significantly higher degradation rate in soil burial conditions compared to PLA, evidenced by larger weight loss and reduction in the molecular weight of the PLA phase. The degradation rate was notably influenced by the morphology of the cellulose phase.

## 1. Introduction

Bio-based, biodegradable polymers—those derived from renewable resources and capable of being broken down by microorganisms in natural environments—are gaining significant interest as a promising alternative to traditional petroleum-based plastics. These materials are particularly appealing due to their reduced environmental impact, both during production, as they are largely independent of fossil carbon sources, and at the end of their lifecycle, where they can degrade naturally, minimizing long-term ecological harm [1,2]. However, to effectively replace conventional plastics, bio-based polymers must exhibit suitable properties, including chemical and physical stability, thermo-mechanical performance, compatibility with standard processing methods, and cost-effectiveness. To achieve these goals, many bio-based polymers are formulated with specific organic or inorganic additives, often resulting in the development of polymeric blends or composite materials. These modifications are essential to enhance their functionality and broaden their potential applications [3,4,5].

Polylactic acid (PLA) stands out as one of the most promising bio-based, biodegradable polymers, primarily due to its low environmental impact, cost-effective production process, and physical and thermo-mechanical properties that are competitive with those of certain commodity plastics. PLA is already widely used in various industrial sectors, including medicine, food packaging, and automotive applications [6,7]. However, the thermo-mechanical properties of PLA present some disadvantages, essentially related to its low ductility, limited thermal stability, and a slow crystallization rate. A wide research effort has been targeting these aspects in recent years, leading to the development of PLA-based materials and blends with interesting properties [5,8,9].

For the specific goal of ductility enhancement, various additives—such as particles, low molecular weight plasticizers, and other polymers—have been incorporated into PLA, resulting in blends and micro- or nano-structured composites. The final properties of these materials are heavily influenced by the interactions between the different phases. In the case of plasticizers, enhancing ductility depends critically on the miscibility of the PLA/plasticizer blend and its long-term stability. This stability is essential to prevent phase separation or the migration of the plasticizer to the material’s surface [10], which can lead to a gradual decline in ductility and toughness, as well as potential contamination of products in contact with the material [11].

Additionally, the intrinsic properties of the plasticizer and its concentration play a crucial role in determining the thermal and mechanical characteristics of the material. Often, enhancing ductility comes at the expense of reduced stiffness and mechanical strength. In this context, research has demonstrated that a synergistic effect between plasticizers and organic or inorganic fillers, and the interactions among them can significantly influence the evolution of the material’s phase structure and properties over time [12,13,14]. This synergy can be exploited to develop ternary systems with finely tuned and balanced performance [15].

Cellulose, a highly abundant natural polymer, has a wide range of applications, including its use as a reinforcing agent in polymer-based composites. Cellulosic fibers are known for their stiffness and strength, making them effective in enhancing the mechanical properties of polymeric materials [16,17]. Additionally, in plasticized composites, the hydroxyl groups on the surface of cellulose can form strong interactions—such as hydrogen bonds or covalent bonds—with the functional groups of the other phases. These interactions, as previously discussed, play a key role in stabilizing the material’s properties over time, further enhancing the performance and durability of the composite [18]. Moreover, the high hydrophilicity and inherent biodegradability of cellulose can accelerate the biodegradation rate of polymeric composites [19,20], a critical factor in evaluating the environmental impact of these materials. Neat PLA degrades slowly under ambient conditions [21,22], which represents an important environmental concern for this polymer. The incorporation of cellulose and cellulosic materials has proved to be an effective way to promote faster degradation kinetics of PLA-based composites [23,24].

Building on these principles, this study focuses on the development of multicomponent PLA-based biocomposites incorporating cellulose as a bio-based filler and adding an oligomeric ester of lactic acid (OLA) for plasticization. To tailor the aspect ratio and surface area of the cellulose filler, the cellulosic components were modified by mechanochemical treatments using a planetary ball mill (BM). Mechanochemistry leverages intense, localized mechanical forces to induce structural changes or even chemical reactions in materials, offering a versatile approach to modifying filler properties for enhanced composite performance [25]. This process is gaining traction as a sustainable “green chemistry” approach, as it often operates under relatively mild conditions and eliminates the need for organic solvents in many cases. Dry ball mill (BM) treatments—conducted without water or other solvents—have been demonstrated to disrupt the crystalline structure of cellulose and break down fibers into a particle-like morphology. This process not only enhances the material’s properties but also aligns with environmentally friendly practices by reducing chemical waste and energy consumption [26]. In contrast, conducting the ball mill (BM) process with a liquid phase (“wet” BM) can lead to the destructuring of cellulosic fibers, potentially yielding high-aspect-ratio fibrils with retained crystallinity [27,28]. This versatility makes the BM process a powerful tool for tailoring the structure and morphology of cellulose and its effects on composite properties by adjusting milling conditions.

From this perspective, in this study cellulose was ball-milled under both dry and wet conditions. In particular, the wet treatment was carried out using the OLA as the only liquid phase, thus avoiding the use of any solvent (including water). This approach can also help promote interactions between the surface functional groups of cellulose and the chemical groups of the OLA.

Binary PLA-cellulose and ternary PLA-cellulose-plasticizer systems were prepared via melt mixing. Thermo-mechanical properties were analyzed and related to the composition and cellulose morphology resulting from the different BM treatments. The stability of the ternary systems was assessed by monitoring property changes over time under controlled storage conditions. Additionally, the water vapor permeability and the degradation rates of the materials were evaluated through soil burial tests to highlight the advantages of the proposed composites in terms of their environmental impact.

## 2. Materials and Methods

### 2.1. Materials

The PLA is a commercial grade (Ingeo 4032D) kindly provided by Natureworks LLC, Plymouth, MN, USA. An oligomeric ester of lactic acid (OLA) characterized in a previous study was selected as plasticizing agent [10]. The cellulose fibers used as reinforcement (Arbocel BWW40, J. Rettenmaier & Söhne GmbH, Rosenberg, Germany) have an average length of about 200 microns and an average diameter of 20 microns.

Before use, the cellulose was dried under vacuum at 90 °C for 24 h, while the PLA and the OLA were dried under vacuum at 60 °C for 24 h.

### 2.2. Mechanochemical Treatments

To investigate the effects of different mechanochemical processing conditions, particularly the influence of a liquid phase, three distinct approaches were designed and implemented:

(a) Dry BM Treatment: Neat cellulose was processed in a planetary ball mill (Retsch PM 100, Retsch GmbH, Haan, Germany) using a 125 mL stainless steel jar and 25 steel spheres (10 mm in diameter). A total of 10 g of cellulose was milled at 600 rpm for 1 h. The resulting material was labeled BMCEL.
(b) Short Homogenization with OLA: The BMCEL material was further homogenized with the OLA plasticizer in the ball mill for 30 min. The process used the same 125 mL steel jar and 25 steel spheres, but at a reduced speed of 400 rpm. The resulting product was coded as OLA/BMCEL BM30′.
(c) Wet BM Treatment: Neat cellulose was milled in the presence of OLA for 2 h or 4 h, under the same conditions as above. This approach mimics a “wet” BM treatment, as OLA acts as a liquid phase. The resulting materials were labeled OLA/BWW40 BM2h and OLA/BWW40 BM4h, respectively.

In all cases, the cellulose-to-OLA ratio was maintained at 1:0.8 to ensure that the final composites had a PLA/OLA weight ratio of 80:20 and a fixed cellulose content of 20 wt%. This systematic approach allowed for a detailed exploration of how different mechanochemical treatments influence the properties of the resulting composites.

### 2.3. Preparation of PLA-Based Composites

For the binary systems, PLA composites containing increasing amounts of ball-milled cellulose (BMCEL) (10, 20, and 30 wt%) were prepared using the first approach described above. These composites were produced by melt mixing at 170 °C in a Brabender Plastograph EC batch mixer, equipped with a heated mixing chamber and two counter rotating blades. In detail, a proper amount of pre-dried PLA pellets was inserted into the heated mixing chamber and allowed to melt for 1 min, then the pre-dried cellulosic material was added and the mixing process was carried out at a rotation speed of 60 rpm for further 7 min. As a reference, a PLA composite with 20 wt% of untreated fibrous cellulose (BWW40) was also prepared under the same conditions.

For the ternary systems, the cellulose/OLA mixtures prepared using the second and third approaches (OLA/BMCEL BM30′, OLA/BWW40 BM2h, and OLA/BWW40 BM4h) were melt-mixed with PLA under the same conditions as the PLA/BMCEL composites, that is, inserting the PLA pellets first in the mixing chamber and then adding the OLA/cellulose mixtures after 1 min to continue with the mixing.

After melt mixing, all materials were recovered, pelletized, and compression-molded at 170 °C under 50 bar pressure for a total of 4 min. This process yielded molded films with a thickness of approximately 200 µm. The compositions and codes of the prepared composites are summarized in Table 1.

### 2.4. Aging

The aging study was carried out via storing films at 25 ± 0.1 °C and 50 ± 1% RH in a Climacell climatic chamber. Samples were tested at regular intervals, up to a maximum of 16 weeks of aging.

### 2.5. Techniques

For the morphological characterization of BMCEL (and BWW40 neat cellulose, for comparison), a scanning electron microscope SEM FEI Quanta 200 FEG (Thermo Fisher Scientific Inc., Waltham, MA, USA), was used. In the case of the OLA/BMCEL and OLA/BWW40 mixtures, SEM analysis was performed after repeated cycles of washing in acetone to remove the non-interacting plasticizer phase. The morphology of the composites was analyzed by carrying out SEM analyses on cryogenic fracture surfaces. Before the SEM analyses, all samples were mounted on aluminum stubs and coated with a layer of Au-Pd alloy with an Emitech K575x sputter coater (Quorum Technologies Ltd., Ashford, UK).

Differential scanning calorimetric (DSC) analyses were carried out using a TA-Q2000 instrument (TA Instrument, New Castle, DE, USA). A ≈5 mg sample of each material was analyzed using a non-hermetic aluminum pan with a heating/cooling rate of 20 °C/min. DSC runs for binary composites were carried out in the temperature range from −20 to 190 °C, while for plasticized materials the range explored spanned from −70 to 190 °C. The main parameters calculated from DSC data were the glass transition temperature, the temperatures corresponding to cold crystallization and to the solid-melt transition and the associated transition enthalpies. For each system, the degree of crystallinity referred to the PLA fraction was calculated considering that the enthalpy of fusion for fully crystalline PLA is equal to 93.6 J/g [29].

Thermogravimetric (TGA) analyses were performed on a Perkin Elmer Pyris One analyzer (Perkin Elmer, Shelton, CT, USA), using platinum crucibles and heating the samples from 40 to 700 °C in air at a heating rate of 20 °C/min.

Tensile tests were performed on an Instron 5564 testing machine (ITW Inc., Glenview, IL, USA), using dumbbell specimens with a section of about 0.8 mm^2^, a grip separation rate of 10 mm/min and a distance between grips of 28 mm. Tests were carried out at a temperature of 27 ± 1 °C, controlled by means of a Instron 3119 chamber. For each material, the elastic modulus, stress at break, and ultimate elongation were calculated as average values over at least 6 specimens.

Water vapor transmission tests were performed according to the ASTM E96 standard [30], measuring the weight variations of cups filled with water and sealed with films with a predefined area of the materials under testing exposed to fixed temperature and relative humidity conditions (25 ± 0.1 °C, 50 ± 1% RH). The water vapor transmission rate (WVTR) was calculated from the slope of the weight loss vs. time curve at the steady state.

Soil burial tests were performed by using a commercial cultivation soil (Organic carbon 40%; electrical conductivity 1.0 dS/m; dry bulk density 950 kg/m^2^; total porosity 85.0% *v*/*v*; pH 7). Three samples of each formulation were buried at a depth of about 5 cm in a container stored at room temperature, adding water at regular intervals to maintain a high humidity. Samples were recovered and cleaned with filter paper at intervals, and their weight was recorded after a stabilization time of at least 5 h under vacuum.

Gel Permeation Chromatography (GPC) analyses were performed in CHCl_3_ (Romil Ltd., Cambridge, UK) at 35 °C, with a flow rate of 0.8 mL/min and a runtime of 45 min, by means of a Malvern-Viscotek GPC MAX/TDA 305 triple detector array (Malvern Panalytical Ltd., Malvern, UK). The system was equipped with a precolumn and two Phenogel (Phenomenex, Torrance, CA, USA) columns, with exclusion limits of 10^6^ and 10^3^ Da. Samples were dissolved in chloroform and filtered through a 0.22 μm PTFE filter before injection (100 µL). Universal calibration was carried out using polystyrene standards in the molecular weight range from 1700 kDa to 1290 MDa.

## 3. Results and Discussion

### 3.1. Morphology of Cellulose After Mechanochemical Treatments

**Figure 1:**
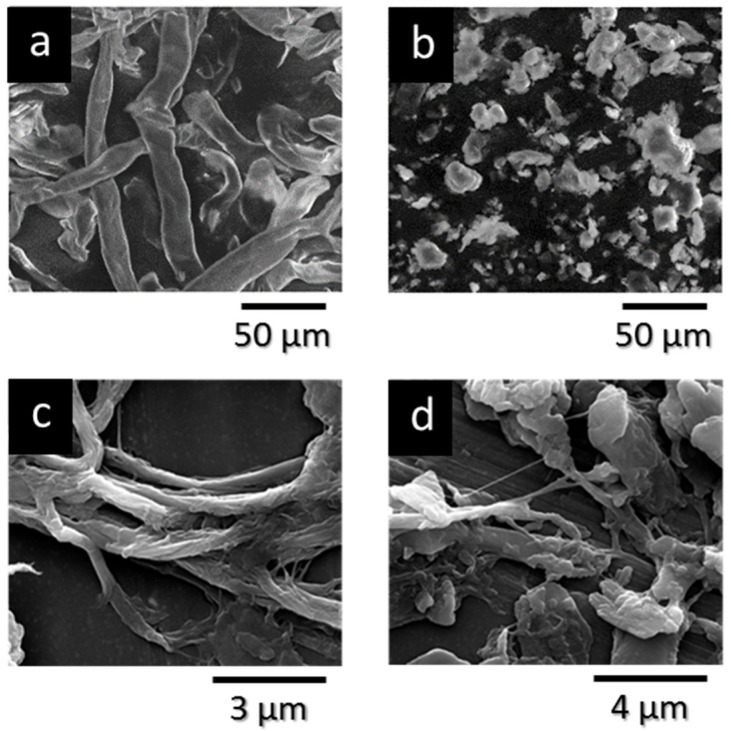
SEM micrographs of (**a**) commercial BWW40 cellulose; (**b**) BWW40 after dry ball milling treatment (BMCEL); (**c**) OLA/BWW40 system treated for 2 h in ball mill; (**d**) OLA/BWW40 system treated for 4 h in ball mill.

SEM analysis was carried out to assess the morphological changes in cellulose resulting from different ball mill (BM) treatments. The micrographs, as depicted in Figure 1, reveal significant alterations in cellulose structure. Untreated cellulose exhibits its typical fibrous morphology (Figure 1a), while dry BM treatment causes extensive fragmentation, resulting in a particle-like appearance (Figure 1b). In contrast, “wet” BM treatment, conducted in the presence of the liquid plasticizer (OLA), partially deconstructs the original cellulose fibers, producing fibrils with thicknesses ranging from 100 to 400 nm and lengths spanning one to several micrometers. These fibrils, however, remain partially embedded within the original fiber structure (Figure 1c,d).

These morphological observations highlight the versatility of BM treatments in tailoring cellulose morphology, which is expected to influence the properties of cellulose-based materials.

### 3.2. Unaged PLA-Based Composites

Before conducting any analyses, the materials were conditioned in a climatic chamber for 24 h at controlled temperature and humidity, as detailed in the experimental section. These samples were designated as unaged samples for reference.

**Figure 2:**
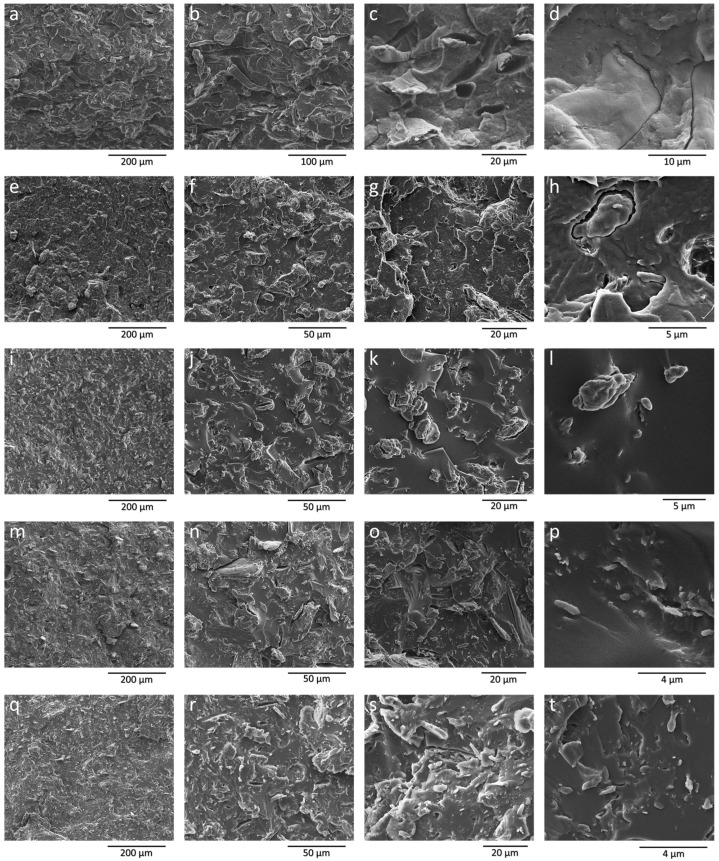
SEM images of PLA + 20BWW40 (**a**–**d**), PLA + 20BMCEL (**e**–**h**), PLA + (OLA/BMCEL) BM30′ (**i**–**l**), PLA + (OLA/BWW40) BM2h (**m**–**p**) PLA + (OLA/BWW40) BM4h (**q**–**t**).

The cryogenic fracture surfaces of all composite materials with a fixed cellulose content of 20% were examined using SEM, and the resulting micrographs are presented in Figure 2. These images provide insights into the microstructure and interfacial interactions between the PLA matrix and cellulose filler, offering a basis for understanding the material’s mechanical and thermal behavior.

In the sample containing untreated BWW40 cellulose (Figure 2a–d), the morphology of the cellulose remains largely unaffected by the melt mixing process, retaining its characteristic ribbon-like shape with fiber lengths reaching up to hundreds of micrometers. Similarly, in composites prepared with dry ball-milled cellulose (BMCEL) (Figure 2e–h), the spheroidal particle structure resulting from the dry BM process is evident, with particle sizes ranging from a few micrometers to 10–20 µm. Both systems show relatively good dispersion of the filler, with no significant aggregation observed. However, high-magnification micrographs reveal poor adhesion at the cellulose/PLA interface, with extensive debonding occurring during the fracture process (Figure 2c,d,g,h).

In the ternary systems, notable differences were observed. For the PLA + (OLA/BMCEL) BM30′ composite (Figure 2i–l), the morphology of BMCEL was not significantly altered by the short homogenization process with OLA, and no defibrillation was evidenced even at high magnification. In contrast, composites containing wet-milled cellulose (OLA/BWW40 BM2h and OLA/BWW40 BM4h) exhibited partial defibrillation, as previously noted (Figure 1). High-magnification images revealed numerous sub-micrometric structures (Figure 2o,p,s,t), indicating the release and dispersion of cellulose fibrils into the matrix. However, the aspect ratio of these fibrils was lower than that observed after wet BM, likely due to fragmentation during the melt mixing process. Additionally, residual micrometric cellulose fibers, damaged but not fully defibrillated by the BM process, were still present in both wet-milled systems.

Adhesion between cellulose and the plasticized PLA phase in the ternary systems appeared improved compared to the binary systems. While some debonding was still observed around larger fibers (Figure 2n,r), cellulose was generally well embedded in the polymer phase after cryogenic fracture. This enhanced adhesion can be attributed to the presence of OLA, which is miscible with PLA and capable of forming hydrogen bonds between the hydroxyl groups on cellulose surfaces and its ester carbonyl and terminal –OH groups [31]. In this context, OLA could be compared to a compatibilizing agent, promoting stronger interfacial adhesion between the polymeric matrix and the cellulosic fillers.

The calorimetric and thermogravimetric properties of the composites, as determined by DSC and TGA, are summarized in Table 2 and Table 3, respectively. DSC thermograms and TGA curves are reported in the supplementary material (Figures S1 and S2).

The thermal transitions of PLA are not strongly influenced by the presence of cellulose or its morphology in binary composites. The main observed effect is a slight increase in crystallinity induced by the addition of BMCEL (ball-milled cellulose). This can be attributed to the higher surface area and significantly smaller particle size of BMCEL compared to untreated BWW40 cellulose. This finding is in line with literature reports suggesting that in PLA/cellulose systems, a nucleating effect is more pronounced when the filler has a submicrometric size, as smaller particles provide more nucleation sites for crystallization [32,33]. An effect of cellulose on PLA crystallization can also be inferred by observing the narrowing of the crystallization peak with respect to PLA, the splitting of the melting peak, and the appearance of a low temperature melting peak (Figure S1). In ternary systems, the absence of any thermal transition associated with the OLA phase (which has a glass transition temperature, Tg, of −43.8 °C) confirms good miscibility between PLA and the plasticizer. The incorporation of the plasticizer leads to a significant reduction in both the glass transition temperature (Tg) and cold crystallization temperature (Tcc) compared to neat PLA and binary, unplasticized composites. Additionally, plasticized composites exhibit a higher degree of crystallinity. These effects can be attributed to the plasticizer’s ability to enhance the mobility of polymer chains, facilitating chain rearrangement. This increased mobility not only lowers the glass transition temperature but also accelerates the crystallization process, resulting in a higher crystallinity degree in the plasticized materials [34].

On the other hand, all plasticized materials exhibited a melting temperature lower than that of neat PLA and binary composites, indicating that the plasticizer influences the crystalline phase of PLA [35]. This behavior has been previously observed in PLA/OLA systems and is linked to PLA’s ability to form multiple crystalline phases with varying defect concentrations, which in turn results in different melting temperatures. Specifically, the melting temperature of plasticized systems closely aligns with the lower melting signal occasionally seen in neat PLA samples [36], which is attributed to the melting of metastable, defective crystals. It is therefore inferred that while OLA enhances the mobility of PLA chains and accelerates the crystallization rate, it also promotes the formation of more defective crystals and lower melting structures during the crystallization process [37].

It is noteworthy that the thermal parameters and phenomena observed in ternary systems are entirely independent of the ball milling conditions (whether “dry” or “wet”) and the duration of cellulose pre-treatment. This indicates that the influence of the plasticizer overshadows any effects arising from the different cellulose morphologies (particle-like or partially defibrillated) achieved through these processes. As previously observed in other ternary systems, when the plasticizer molecules exhibit a strong affinity for the filler surface, they tend to adsorb onto it, effectively “masking” the particle surface. This reduces the filler’s efficiency in nucleating PLA crystallization, further emphasizing the dominant role of the plasticizer in shaping the thermal behavior of the composite [13].

As concerning thermogravimetric properties, PLA exhibits one degradation step starting at 271 °C. Binary composite materials show a small weight loss phenomenon at lower temperatures (between 110 and 200 °C) and an additional weight loss step positioned at a higher temperature (Figure S2) than is often observed in cellulosic materials and corresponds to the combustion of the char produced by the main cellulose degradation process. It is interesting to observe that in all binary composites, both the temperature of the onset of the degradation (conventionally taken as the temperature corresponding to 1% of the weight loss) and the temperature of maximum weight loss are shifted to higher temperatures, indicating a general improvement of the thermal stability of composites with respect to PLA. This finding is somewhat expected, as the thermal degradation of cellulose is usually reported at about 300 °C, higher than PLA. Indeed, the composites containing unmodified cellulose, PLA + 20BWW40, show higher thermal stability because crystalline cellulose is more stable than ball-milled, amorphized cellulose (BMCEL).

In plasticized materials, by contrast, the thermal stability is reduced as a consequence of the presence of OLA. The low molecular weight additive is in fact susceptible to degradation in presence of the humidity absorbed by the composite samples that were stored at 50% RH. This phenomenon is known but was taken under control during the melt mixing process, as all materials were carefully dried before the process to avoid any degradation.

The mechanical parameters of all prepared materials as obtained by tensile tests are reported in Table 4, while examples of the stress-strain curves are reported in Figure S3. These results provide further insights into the influence of cellulose morphology, OLA plasticization, and interfacial interactions on the overall performance of the materials.

Regarding the mechanical properties of unaged materials (Table 4), the results are best analyzed by separating binary and ternary systems. In binary systems, the addition of cellulose increases the elastic modulus compared to neat PLA. However, this higher modulus is accompanied by a reduction in ultimate elongation and strength. This decline in performance suggests a weak interface between cellulose and the PLA matrix, leading to adhesion loss under mechanical stress and premature composite failure. This poor interfacial adhesion, observed for both BWW40 (fibrous cellulose) and BMCEL (particle-like cellulose), was also evidenced in the morphological analyses (Figure 2). Interestingly, the mechanical response of composites containing fibrous cellulose (BWW40) is similar to that of composites with particle-like BMCEL at the same cellulose content (20 wt%). In theory, longer fibers should provide stronger reinforcement, particularly in terms of elastic modulus, which is measured at low deformation and is less sensitive to interfacial adhesion than strength or elongation. The limited reinforcement observed in the PLA + 20 BWW40 system is likely due to the lack of fiber orientation (Figure 2a–c), which reduces the reinforcing efficiency of the fibers. These findings highlight the critical role of interfacial adhesion and filler orientation in determining the mechanical performance of cellulose-reinforced PLA composites [38].

In ternary systems, the inclusion of the plasticizer (OLA) significantly altered the mechanical behavior. Plasticization markedly enhanced the ductility of the PLA matrix, resulting in composites with ultimate elongation exceeding 100%. The addition of the cellulosic phase further improved the mechanical properties, leading to a notable increase in both elastic modulus and strength compared to binary PLA/OLA systems of similar composition, as reported in previous studies. This demonstrates that the combination of plasticizer and cellulose not only enhances flexibility but also reinforces the material, achieving a balance between ductility and mechanical strength [14].

Moreover, the ductility of plasticized composites is significantly influenced by the morphology of the cellulosic phase. BMCEL, characterized by its low aspect ratio, enhances ductility, resulting in higher ultimate elongation for the PLA + (OLA/BMCEL) BM30′ composite compared to materials containing wet BM cellulose [19]. However, despite the differences in cellulose morphology affecting ductility, no substantial variations were observed in strength and stiffness. This behavior can be attributed to the relatively low aspect ratio of the fibrils produced during the wet BM process, as well as the presence of residual micrometric cellulose fibers in both the PLA + (OLA/BWW40) BM2h and PLA + (OLA/BWW40) BM4h materials (Figure 2n–p,r–t). These larger particles act as defect points that can initiate premature fracture during deformation, limiting improvements in strength and stiffness despite the enhanced ductility provided by the plasticizer.

Transport properties, particularly relevant for potential applications of PLA-based composites in the packaging sector, were evaluated through the water vapor transmission rate (WVTR), measured using the cup method (ASTM E96), as detailed in the Materials and Methods section. The calculated WVTR values are presented in Table 5. WVTR increases in all composite materials compared to neat PLA, primarily due to the high hydrophilicity of cellulose. In binary formulations, WVTR is roughly proportional to the cellulose content. Notably, plasticized composites exhibit higher WVTR than binary systems with the same cellulose content (20%), underscoring the influence of the plasticizer on the transport properties of ternary systems. OLA plasticizers, being more hydrophilic than PLA, enhance water absorption and accelerate water transport through the material. Additionally, plasticization increases the free volume within the polymer matrix, which can lead to higher solubility and permeation of gases and vapors [39]. Furthermore, the cellulose fibrils produced by wet ball milling (PLA + (OLA/BWW40) BM2h and PLA + (OLA/BWW40) BM4h samples) resulted in the highest increase in WVTR. This is attributed to their greater available surface area and higher hydrophilicity, which further promotes water vapor transmission.

The water permeation properties of these materials can have important consequences in relation to the soil burial degradation tests, as discussed in Section 3.3.

### 3.3. Aging and Stability of Properties

**Figure 3:**
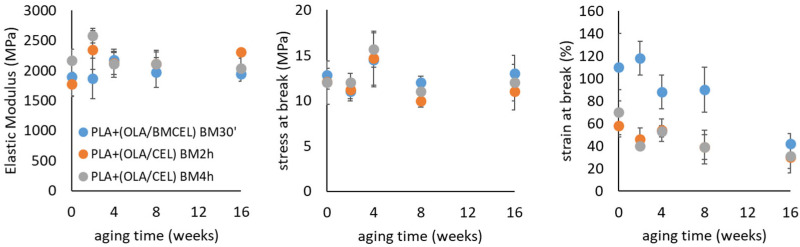
Mechanical parameters recorded on plasticized composites upon aging.

For plasticized materials, mechanical properties were monitored at regular intervals during storage under controlled conditions to evaluate changes caused by the evolution of the phase structure, such as potential clustering or segregation of the plasticizer over time. The trends in the mechanical parameters over time are shown in Figure 3, while numerical values are reported in Table S1 in the supplementary material. A key observation is that the elastic modulus and ultimate tensile strength remained relatively stable for all materials up to the maximum aging time of 16 weeks, even though some fluctuation was recorded. In contrast, the elongation at break exhibited more significant variations during aging, particularly for the plasticized material containing BMCEL. This sample initially had an elongation at break of 110% before aging, which progressively decreased to approximately 40% after 16 weeks. In contrast, plasticized composites containing partially defibrillated cellulose showed a much smaller reduction in ductility, from about 60% to around 40%.

The ductility of plasticized materials is highly sensitive to the development of heterogeneities caused by partial segregation or migration of the plasticizer. Such phase separation can lead to the formation of unplasticized regions within the material, resulting in embrittlement. In PLA/OLA binary systems, for example, a significant reduction in ductility was observed after just 1 week of aging, with overall stability limited to 8 weeks [11]. In ternary systems like the plasticized composites studied here, the presence of filler particles has been shown to influence the phase separation behavior of the plasticizer. Specifically, a stabilization of properties has been achieved when strong interactions between the plasticizer and filler surfaces occur, leading to the adsorption of plasticizer molecules. This finding is similar to what has been observed in systems such as PLA-OLA-calcium carbonate composites.

As previously noted, OLA molecules are expected to form strong hydrogen bonds with the hydroxyl groups on the cellulose surface, which explains the improved stability of ternary systems compared to unfilled PLA/OLA systems. The higher surface area of fibrillated cellulose further enhances this stability by providing more interaction sites for OLA molecules. This interaction helps mitigate plasticizer migration and phase separation, thereby preserving the ductility and mechanical properties of the composites over time.

### 3.4. Soil Burial Degradation Test

**Figure 4:**
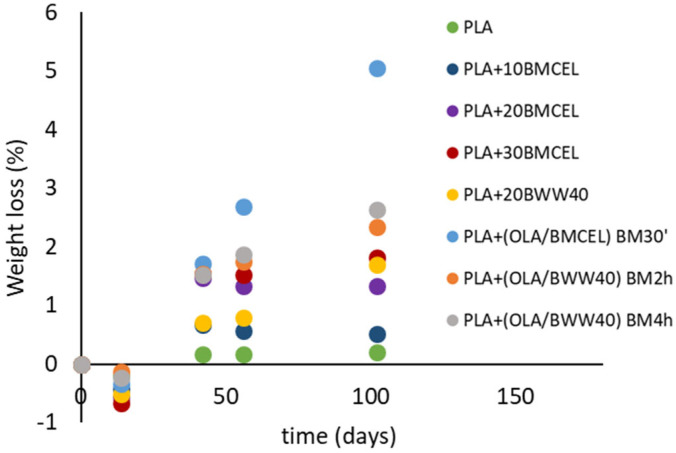
Soil burial tests data recorded on the prepared materials. Maximum error is estimated as ±0.5%.

To assess the impact of the plasticizer and cellulose with varying morphologies on the environmental degradation behavior of the materials, a soil degradation test was conducted. Specimens of all samples were buried in standard cultivation soil, and their weight loss was monitored over time. This test simulates the uncontrolled dispersion of materials in the environment, replicating one of the scenarios of plastic waste mismanagement that can lead to long-term environmental consequences. The weight loss data collected over time are presented in Figure 4.

First, it is important to note that all samples exhibited a slight increase in weight at the initial testing time, resulting in a negative weight loss. This observation is not unexpected and is attributed to the absorption of water by the samples when placed in contact with moist soil. This absorbed water is not entirely removed by the vacuum treatment used for weight stabilization. The recorded value can be considered a nearly constant offset, which is eventually overshadowed by the weight loss observed at longer burial times. A general trend can be identified in the weight loss versus time behavior of the composites: while neat PLA shows negligible weight loss over the tested period, the addition of cellulose significantly accelerates degradation. Furthermore, plasticized materials degrade even faster than their non-plasticized counterparts. The soil degradation of PLA-based materials is a complex process involving hydrolysis and microbial activity [40]. However, it is widely reported that water uptake is the primary factor influencing the degradation rate. This explains why materials with higher hydrophilicity, such as those containing cellulose and plasticizers, exhibit faster degradation in soil burial conditions [24,41].

The presence of cellulose significantly increases the hydrophilicity of the composites compared to neat PLA, as also evidenced by the WVTR tests, leading to a degradation rate that is roughly proportional to the cellulose content. In the case of ternary systems, the plasticizer further enhances water uptake (as discussed in Section 3.1 and shown in Table 5), resulting in an even greater increase in weight loss during soil burial, as illustrated in Figure 4. Among the tested samples, PLA + (BMCEL/OLA) BM30′ exhibited the fastest rate of weight loss. This result can be attributed to the fact that BMCEL not only has a large surface area but also a significantly reduced crystallinity [26], making it more susceptible to degradation compared to native cellulose fibers and fibrils. These factors collectively contribute to the accelerated degradation observed in this material [23,42].

To further investigate the degradation pathway and assess the impact of soil burial on PLA, the molecular weight of the PLA fraction in samples containing 20 wt% cellulose was measured before burial and after 56 and 102 days of burial, as detailed in Table 6. An initial observation was that the number-average molecular weight (Mn) and weight-average molecular weight (Mw) of the composites before burial were lower than those of neat PLA. This reduction was likely due to the release of residual water from cellulose during melt mixing, which can induce some degree of PLA hydrolysis. Despite thorough drying under vacuum at 100 °C, additional water may still be released from cellulose at the higher processing temperature of 170 °C, contributing to this effect. These findings highlight the sensitivity of PLA to hydrolysis, even during processing, and underscore the importance of controlling moisture content in cellulose-reinforced composites to minimize premature degradation.

Concerning the trend of molecular weight as a function of burial, PLA, as expected [22], did not show a significant change in either Mn or Mw (the error of GPC measurement can be estimated as ±10%). The binary composite materials showed some limited signs of hydrolysis, in particular at the longest burial time, while plasticized composites showed a relevant degradation of the PLA phase, with the maximum Mw decrease accounting for 37% of the initial value.

These findings confirm that:

− Cellulose accelerates PLA degradation: The presence of cellulose increases the degradation rate of PLA, at least under the conditions used in this study, by promoting water uptake. This is consistent with the observed increase in hydrophilicity and water vapor transmission rate (WVTR) in cellulose-containing composites.
− Plasticizer significantly enhances degradation: The inclusion of a low molecular weight plasticizer (OLA) has a pronounced effect on the degradation rate of PLA, leading to a substantial reduction in both number-average (Mn) and weight-average (Mw) molecular weights. This is attributed to the plasticizer’s ability to increase water absorption and facilitate hydrolysis.

These results highlight a potential strategy for modulating the degradation rate of PLA-based materials by carefully selecting additives with specific properties and employing mechanochemical pretreatments. PLA is known for its slow degradation rate in ambient conditions, as evidenced by the soil burial test and other long-term degradation studies [43] (e.g., over 12 months).

However, the molecular weight recorded on the PLA phase of composites after the longest burial time (102 days) remained relatively high, indicating that complete degradation and fragmentation of the polymer down to soluble oligomeric species had not yet occurred. This suggests that the largest part of the weight loss observed during the burial test, particularly for the PLA + (OLA/BMCEL) BM30′ sample, should be attributed to the degradation of the amorphous cellulosic phase (BMCEL) rather than to the degradation of the PLA matrix.

These insights underscore the complex interplay between cellulose, plasticizers, and PLA in determining the degradation behavior of these composites, offering valuable guidance for designing materials with tailored environmental performance.

## 4. Conclusions

In this study, PLA-based composites incorporating a plasticizer (an oligomeric ester of lactic acid, OLA) and cellulose with varying morphologies as reinforcement were developed. Cellulose was subjected to ball mill (BM) pretreatments under different conditions (dry and wet) to modify its structure and morphology. The effects of these modifications on the thermo-mechanical properties, water vapor permeability, and degradation rate of the composites were investigated. Unplasticized composites were also prepared and tested for comparison.

In plasticized composites, the thermo-mechanical properties and their stability over time are closely linked to both the effects of the ball milling treatment on cellulose and the interactions between cellulose and the plasticizer. The inclusion of cellulose, particularly amorphized, particle-like cellulose (BMCEL), helps maintain the ductility imparted by the plasticizer while preventing the significant reduction in elastic modulus and strength often seen in plasticized materials. On the other hand, partially fibrillated cellulose produced through wet ball milling enhances the stability of properties over time, as its larger surface area provides more hydroxyl groups for interaction with OLA, thereby limiting phase separation and migration of the plasticizer.

All composites exhibited increased water vapor permeability, influenced by both the morphology of cellulose and the presence of the low molecular weight OLA. This increased permeability correlated with faster degradation in soil burial conditions, as evidenced by both a greater weight loss over time, essentially due to cellulose degradation, and a reduction in the molecular weight of the PLA phase, evidenced by GPC and not observed in neat PLA. The degradation rate was particularly affected by the morphology of the cellulose phase.

In summary, PLA-based systems containing plasticizers and mechanochemically treated cellulose represent promising biodegradable materials. By modulating cellulose morphology through different treatment conditions and exploiting the synergistic effects of the plasticizer, it is possible to control the mechanical behavior and degradation rate of these materials. This approach addresses environmental concerns associated with the slow degradation of PLA, offering a pathway to design materials with tailored performance and reduced ecological impact. Possible applications of such materials include PLA-based flexible packaging films with enhanced ductility and compostability.

## Figures and Tables

**Table 1:** Composition and codes of all materials.

Sample	PLA/OLA Ratio	Cellulose (wt%)
PLA	100/0	0
PLA + 10BMCEL	100/0	10
PLA + 20BMCEL	100/0	20
PLA + 30BMCEL	100/0	30
PLA + 20BWW40	100/0	20
PLA + (OLA/BMCEL) BM30′	80/20	20
PLA + (OLA/BWW40) BM2h	80/20	20
PLA + (OLA/BWW40) BM4h	80/20	20

**Table 2:** DSC parameters (first heating run): glass transition temperature Tg (°C), cold crystallization temperature Tcc (°C), melting temperature Tm (°C), degree of crystallinity Xc. Experimental errors are estimated as ±1 °C for temperatures and ±2% for crystallinity content.

Sample	Tg (°C)	Tcc (°C)	Tm (°C)	Xc (%)
PLA	55	106	167	5
PLA + 10BMCEL	55	106	168	7
PLA + 20BMCEL	53	107	168	10
PLA + 30BMCEL	55	104	169	8
PLA + 20BWW40	55	108	169	5
PLA + (OLA/BMCEL) BM30′	31	80	159	17
PLA + (OLA/BWW40) BM2h	31	79	160	18
PLA + (OLA/BWW40) BM4h	31	79	160	17

**Table 3:** Thermogravimetric parameters obtained from TGA analysis: Temperature corresponding to the 1% of weight loss (T_1%_), Temperature corresponding to the maximum weight loss rate (T_max_).

Sample	T_1%_ (°C)	T_max_ (°C)
PLA	271	344
PLA + 10BMCEL	297	355
PLA + 20BMCEL	288	349
PLA + 30BMCEL	287	356
PLA + 20BWW40	302	367
PLA + (OLA/BMCEL) BM30′	171	315
PLA + (OLA/BWW40) BM2h	178	359
PLA + (OLA/BWW40) BM4h	172	360

**Table 4:** Elastic modulus E (MPa), stress at break (σb) and ultimate elongation εb (%) as calculated from tensile tests.

Sample	E (MPa)	εb (%)	σb (%)
PLA	2750 ± 90	4.0 ± 0.1	56 ± 1
PLA + 10BMCEL	3000 ± 100	2.1 ± 0.3	48 ± 4
PLA + 20BMCEL	3100 ± 100	1.5 ± 0.3	39 ± 4
PLA + 30BMCEL	3100 ± 100	1.2 ± 0.1	28 ± 5
PLA + 20BWW40	3200 ± 70	1.2 ± 0.3	38 ± 5
PLA + (OLA/BMCEL) BM30′	1900 ± 200	110 ± 30	13 ± 1
PLA + (OLA/BWW40) BM2h	1800 ± 200	60 ± 10	12 ± 1
PLA + (OLA/BWW40) BM4h	2200 ± 200	70 ± 20	12 ± 1

**Table 5:** Water Vapor Transmission Rate values recorded on PLA and on all the prepared materials.

Sample	WVTR (g mm/(24 h m^2^))
PLA	2.7
PLA + 10BMCEL	3.9
PLA + 20BMCEL	4.8
PLA + 30BMCEL	7.4
PLA + 20BWW40	4.7
PLA + (OLA/BMCEL) BM30′	5.6
PLA + (OLA/BWW40) BM2h	7.0
PLA + (OLA/BWW40) BM4h	7.4

**Table 6:** Number average (Mn) and weight average (Mw) molecular weight of the PLA fraction in the materials containing 20 wt% of cellulose and subjected to soil burial. Measurements were performed after 0 days (reference, t_0_), 56 days (t_56_) and 102 days (t_102_) of burial. Values are expressed in Daltons, in parentheses are reported the % values with respect to t_0_.

Sample	t_0_		t_56_		t_102_	
	Mn	Mw	Mn	Mw	Mn	Mw
PLA	28,576 (100)	81,841 (100)	29,723 (104.0)	81,568 (99.7)	31,635 (110.7)	75,130 (91.8)
PLA + 20BMCEL	21,139 (100)	47,109 (100)	17,986 (85.1)	44,715 (94.9)	17,825 (84.3)	42,153 (89.5)
PLA + 20BWW40	20,570 (100)	45,594 (100)	20,013 (97.3)	45,665 (100.2)	16,545 (80.4)	41,141 (90.2)
PLA + (OLA/BMCEL) BM30′	18,038 (100)	36,297 (100)	17,647 (97.8)	33,559 (92.5)	14,281 (79.2)	27,097 (74.7)
PLA + (OLA/BWW40) BM2h	24,065 (100)	61,426 (100)	20,445 (85.0)	43,660 (71.1)	19,937 (82.8)	38,646 (62.9)
PLA + (OLA/BWW40) BM2h	25,123 (100)	51,799 (100)	24,252 (96.5)	45,626 (88.1)	18,315 (72.9)	37,932 (73.2)

## Data Availability

The original contributions presented in this study are included in the article/Supplementary Material. Further inquiries can be directed to the corresponding authors.

## Supplementary Materials

The following supporting information can be downloaded at: https://www.mdpi.com/article/10.3390/polym17152127/s1, Figure S1: DSC thermograms of the materials prepared (1st heat): PLA (a); PLA + 10BMCEL (b); PLA + 20BMCEL (c); PLA + 30BMCEL (d); PLA + 20BWW40 (e); PLA + (OLA/BMCEL) BM30′ (f); PLA + (OLA/BWW40) BM2h (g); PLA + (OLA/BWW40) BM4h (h); Figure S2: TGA weight loss curves of the materials prepared: PLA (a); PLA + 10BMCEL (b); PLA + 20BMCEL (c); PLA + 30BMCEL (d); PLA + 20BWW40 (e); PLA + (OLA/BMCEL) BM30′ (f); PLA + (OLA/BWW40) BM2h (g); PLA + (OLA/BWW40) BM4h (h); Figure S3: Examples of tensile stress-strain curves recorded on PLA and on the composites: PLA (a); PLA+10BMCEL (b); PLA + 20BMCEL (c); PLA + 30BMCEL (d); PLA + 20BWW40 (e); PLA + (OLA/BMCEL) BM30′ (f); PLA + (OLA/BWW40) BM2h (g); PLA + (OLA/BWW40) BM4h (h). Curves of brittle samples (a–e) are reported in a single graph with a 1% shift on the strain axis; Table S1: Elastic modulus E (MPa), stress at break (σb) and ultimate elongation εb (%) as a function of aging time.
