# Supplementary material for: Enhancement of Stability Towards Aging and Soil Degradation Rate of Plasticized Poly(lactic Acid) Composites Containing Ball-Milled Cellulose
Source: Polymers (Basel). 2025 Aug 1;17(15):2127. doi: 10.3390/polym17152127 (PMC12349688; doi:10.3390/polym17152127)
Supplement: Supplementary file 1 [file polymers-17-02127-s001.zip › polymers-3720274-supplementary.pdf]

Supplementary Material

Enhancement of Stability Towards Aging and Soil Degradation Rate of Plasticized Poly(lactic Acid) Composites Containing Ball-Milled Cellulose

Roberta Capuano [1,2], Roberto Avolio [1,*], Rachele Castaldo [1], Mariacristina Cocca [1], Federico Olivieri [1], Gennaro Gentile [1] and Maria Emanuela Errico [1,*]

[1] Institute of Polymers, Composites and Biomaterials, National Research Council of Italy (IPCB-CNR), Via Campi Flegrei 34, 80078 Pozzuoli, Italy; roberta.cap2012@gmail.com (R.C.); rachele.castaldo@cnr.it (R.C.); mariacristina.cocca@cnr.it (M.C.); federico.olivieri@cnr.it (F.O.); gennaro.gentile@cnr.it (G.G.)

[2] Department of Mechanical and Industrial Engineering—DIMI, University of Brescia, Via Branze 38, 25121 Brescia, Italy

* Correspondence: roberto.avolio@cnr.it (R.A.); mariaemanuela.errico@cnr.it (M.E.E.)

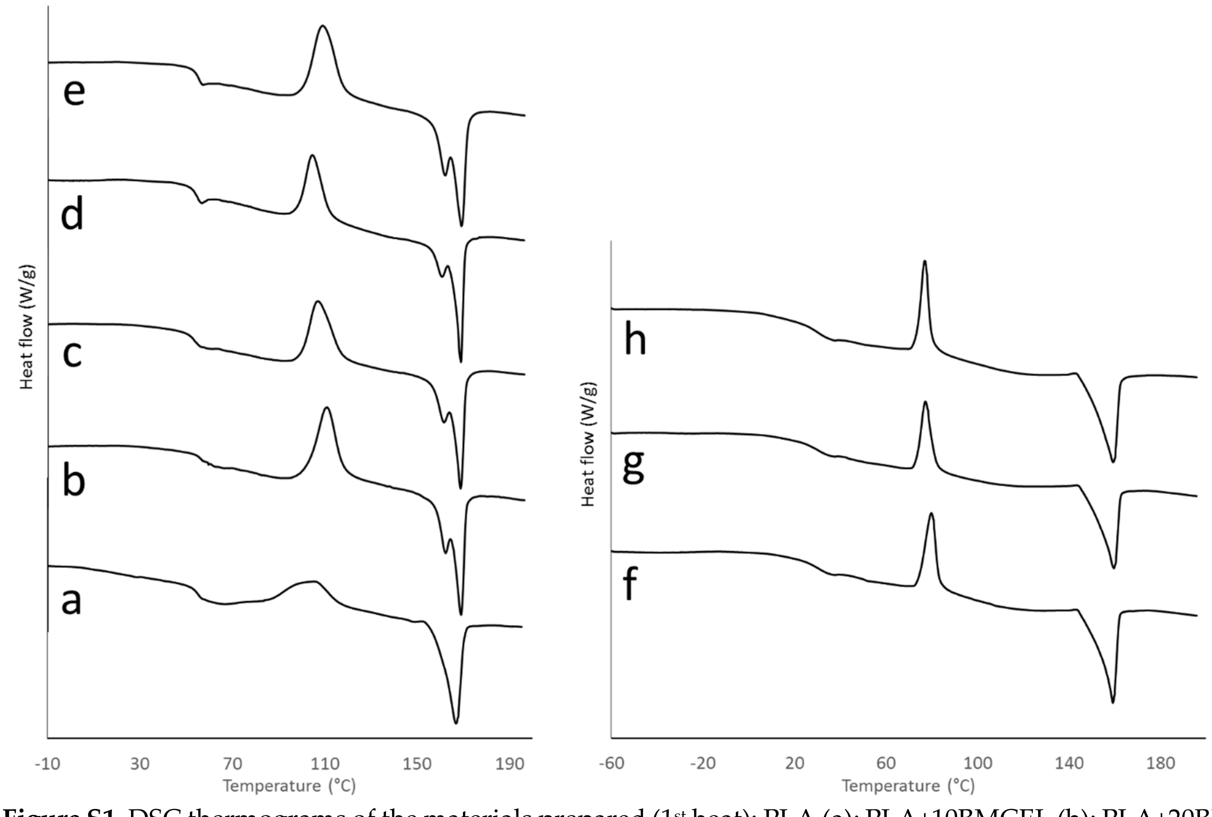

Figure S1. DSC thermograms of the materials prepared (1st heat): PLA (a); PLA+10BMCEL (b); PLA+20BMCEL (c); PLA+30BMCEL (d); PLA+20BWW40 (e); PLA+(OLA/BMCEL) BM30′ (f); PLA+(OLA/BWW40) BM2h (g); PLA+(OLA/BWW40) BM4h (h).

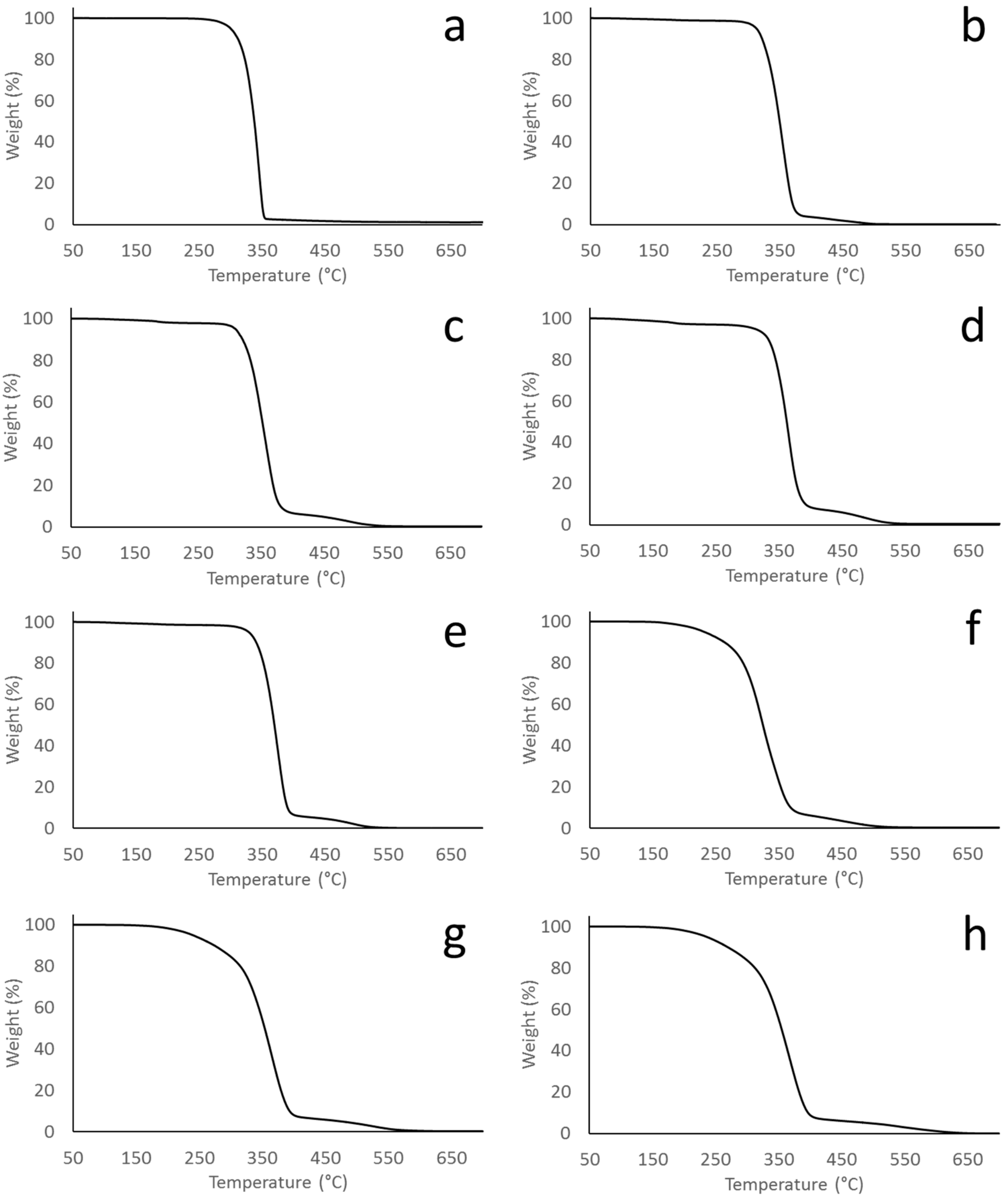

Figure S2. TGA weight loss curves of the materials prepared: PLA (a); PLA+10BMCEL (b); PLA+20BMCEL (c); PLA+30BMCEL (d); PLA+20BWW40 (e); PLA+(OLA/BMCEL) BM30′ (f); PLA+(OLA/BWW40) BM2h (g); PLA+(OLA/BWW40) BM4h (h).

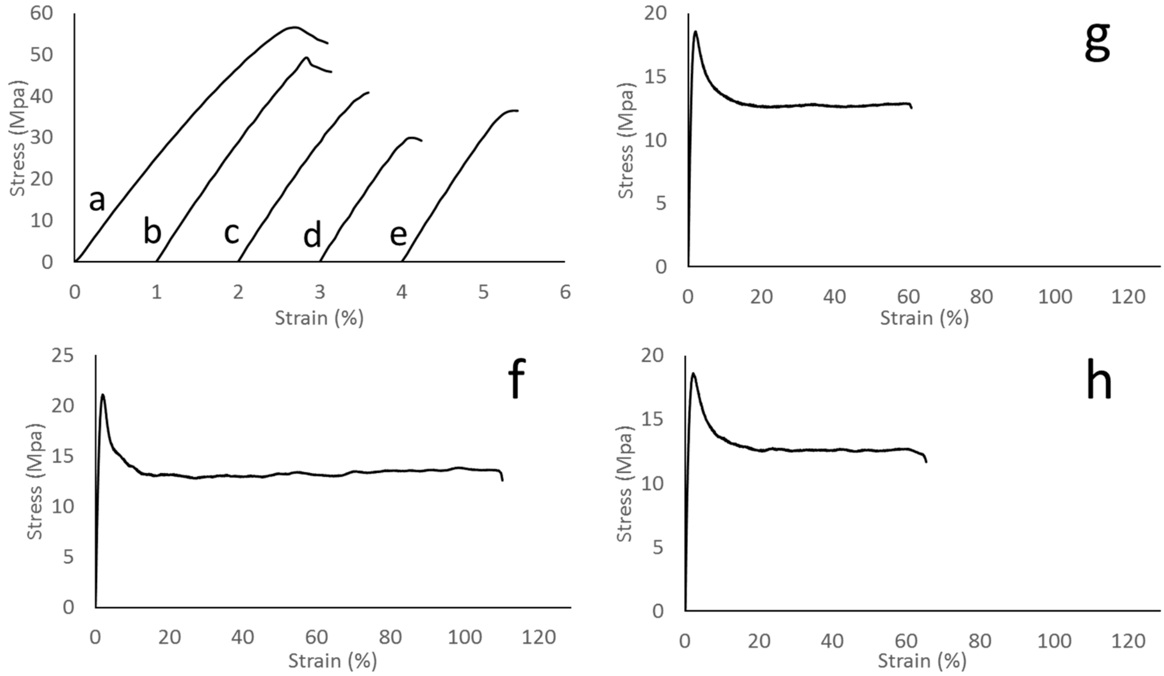

Figure S3. Examples of tensile stress-strain curves recorded on PLA and on the composites: PLA (a); PLA+10BMCEL (b); PLA+20BMCEL (c); PLA+30BMCEL (d); PLA+20BWW40 (e); PLA+(OLA/BMCEL) BM30′ (f); PLA+(OLA/BWW40) BM2h (g); PLA+(OLA/BWW40) BM4h (h). Curves of brittle samples (a-e) are reported in a single graph with a 1% shift on the strain axis.

Table S1. Elastic modulus E (MPa), stress at break (σb) and ultimate elongation εb (%) as a function of aging time

Sample	Aging time (weeks)	E (MPa)	εb (%)	σb (%)
PLA+(OLA/BMCEL) BM30′	0	1900 ± 200	110 ± 30	13 ± 1
	2	1900 ± 300	120 ± 20	11 ± 3
	4	2200 ± 200	90 ± 20	14 ± 1
	8	2000 ± 200	90 ± 10	12 ± 2
	16	1900 ± 100	40 ± 10	13 ± 2
PLA+(OLA/BWW40) BM2h	0	1800 ± 200	60 ± 10	12 ± 1
	2	2300 ± 200	50 ± 10	11 ± 2
	4	2100 ± 300	50 ± 10	15 ± 1
	8	2100 ± 200	40 ± 10	10 ± 2
	16	2300 ± 200	30 ± 10	11 ± 2
PLA+(OLA/BWW40) BM4h	0	2200 ± 200	70 ± 20	12 ± 1
	2	2600 ± 100	40 ± 10	12 ± 2
	4	2100 ± 200	50 ± 20	16 ± 1
	8	2100 ± 200	40 ± 10	11 ± 2
	16	2000 ± 200	30 ± 10	12 ± 1
